# Impact of very long time output variation in the treatment of total marrow irradiation with helical tomotherapy

**DOI:** 10.1186/1748-717X-8-123

**Published:** 2013-05-20

**Authors:** Yutaka Takahashi, Susanta K Hui

**Affiliations:** 1 Masonic Cancer Center, University of Minnesota, 424 Harvard Street SE, Minneapolis, MN 55455, USA; 2 Department of Therapeutic Radiology, University of Minnesota, 424 Harvard Street SE, Minneapolis, MN 55455, USA

**Keywords:** Radiation therapy, Tomotherapy, Total marrow irradiation, Total body irradiation, Very long time output variation

## Abstract

**Background:**

Beam-on time in Total Marrow Irradiation (TMI) delivery with helical tomotherapy is more than 30 minutes. The purpose of this study was to investigate extended time output variation in tomotherapy machine without dose servo system and its impact on the dosimetry of TMI planning.

**Materials and methods:**

The calibration procedures with 1800 seconds delivery were conducted. The slab and cylindrical phantoms were used for static and rotational output variation measurements, respectively. All measurements were performed in 0.1 second interval with an Exradin A1SL ionization chamber (Standard Imaging Inc., Madison, WI, USA) connected to the tomoelectrometer supplied by the manufacture. Simulated TMI treatment planning with a slab phantom was delivered and verified with ion chamber and EDR-2 films.

**Results:**

The static output variations during 30 min averaged −2.9% ± 0.2%, -3.4% ± 0.3%, and −3.4% ± 0.3% at 10 min, 20 min, and 30 min, respectively. The rotational output variations from start averaged −2.5% ± 0.7%, -3.1% ± 0.7%, and −3.5% ± 0.8% at 10 min, 20 min, and 30 min, respectively. The maximum output variation was up to 4.5%. In a TMI planning model, in which beam-on time was over 30 min, planned dose and dose measured with ion chambers in both cranial and caudal sides agreed within 3%. Film measurements in cranial and caudal sides also showed the pass rates of 97.7% and 92.2% with the criteria of 3 mm/3% in gamma analysis.

**Conclusion:**

These results suggest that long TMI delivery by helical tomotherapy, even without dose servo system, does not pose a risk for significant deviations from the original treatment plan regardless of the output variation. However, very long time output variation should be checked before the first treatment.

## Background

Helical tomotherapy is an intensity modulated X-ray therapy modality that is capable of delivering highly conformal dose distributions [1,2]. While the gantry rotates around the patients, the mutileaf collimator opens and closes to produce an intensity modulated beam with variable couch speed. The current treatment couch on the helical tomotherapy unit has a maximum travel length of approximately 150 cm.

These features allow for the delivery of intensity modulated beams for the treatment of very long targets like in the case of total body irradiation (TBI) or total marrow irradiation (TMI), which was first proposed by Hui et al. [3]. Dosimetric and clinical studies have been intensively performed and the feasibility of tomotherapy machine for TBI and TMI has been shown by several groups [4-7].

On the other hand, TBI or TMI deliveries with tomotherapy machine sometimes require more than 30 minutes of beam-on time [3,5,6]. Although the new Dose Control System (DCS) has been offered to the current tomotherapy machine since 2011, most tomotherapy machines worldwide do not have DCS. Very long time output variation is therefore especially important in TBI or TMI planning delivery in the tomotherapy machines without DCS. Although several groups reported the output variations in imaging beam [8] or treatment beam modes [9-11], no report has been published assuming very long time treatment such as TBI, and TMI.

Here we report the 30 minutes’ output variations in static and rotational procedures. Furthermore, the impact of long time output variations on TMI planning delivery was investigated.

## Materials and methods

All measurements were performed with an Exradin A1SL ionization chamber (Standard. Imaging Inc., Madison, WI, USA) connected to the 8-channel electrometer (Tomoelectrometer, Standard. Imaging Inc., Madison, WI, USA) supplied by the manufacturer. This electrometer can operate in two modes: local and software controlled. In the latter mode, the tomotherapy measurement system software (TEMS) is used to collect the measured charge in real time with a sampling time resolution that can be set as low as 100ms. In this way, the ion chamber readings vs. time can be collected throughout the overall radiation delivery. Two phantoms were used: a set of solid water stacks and a cylindrical solid water phantom, both supplied by the manufacturer (Accuray, Inc. Madison, WI, USA). Treatment planning and dose verification analysis were done with TomoTherapy Planning Station (Accuray, Inc., Madison,WI, USA).

### Long time output variation in static beam

The solid water phantom of 55 (length) × 15 (width) × 8 (depth) cm^3^ was used for the measurements with AlSL ion chamber with a volume of 0.053 cc at 1.5 cm depth connected to the tomoelectrometer. Ion chamber readings were collected every 0.1 second. Radiation was delivered for 1800 seconds with the field size of 5 × 40 cm^2^ at a source-to-surface distance (SSD) of 85 cm.

Since the nominal dose rate input in the treatment planning system was defined for a 5 × 40 cm^2^ field size at a distance of 85 cm (isocenter) at a depth of 1.5 cm (SSD = 83.5 cm) [12], the dose in this condition from our measurement geometry was calculated by the following formula.

$$D=M\cdot N_{D,W,60_{\mathrm{Co}}}\cdot k_{\text{Tomo},60_{\mathrm{Co}}}\cdot {\left[\frac{\text{SAD}}{\text{SAD}+d_{\max}}\right]}^{2}$$

where *M* is the fully corrected electrometer reading, measured in a static beam. $N_{D,W,60_{\mathrm{Co}}}$ is the absorbed dose to water calibration factor for the cobalt beam quality and $k_{\text{Tomo},60_{\mathrm{Co}}}$ is the quality conversion factor, which converts a ^60^Co absorbed dose calibration into one suitable for the tomotherapy beam.

The measurements were repeated 3 or 4 times on other days for static and rotational output, respectively.

### Long time output variation in rotational beam

The manufacture supplied cylindrical phantom (TomoPhantom, Accuray, Inc., Madison, WI, USA) was placed on the treatment couch and the ion chamber was inserted at 0.5 cm below from the center of the phantom.

We conducted the procedures of gantry rotation speed of 10 seconds/rotation with the field size of 5 × 40 cm^2^ at a SSD of 70 cm (source-cylindrical phantom center-distance of 85 cm) and radiation was delivered for 1800 seconds. The ion chamber reading was collected every 0.1 second. Because the rotational output oscillates, the average values of each cycle were calculated and plotted.

### TMI planning delivery and dose verification

**Figure 1:**
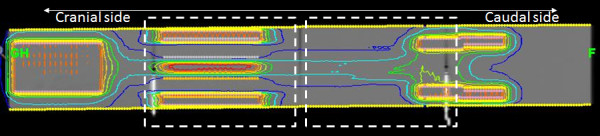
**A coronal plane of simulated TMI planning in the solid water phantom of 110 (length) × 15 (width) × 8 (depth) cm**^**3**^**.** Two chambers and films were put in cranial and caudal sides at the depth of 2 cm and 4 cm, respectively. White dashed rectangular lines indicate the locations of films.

A model TMI planning was conducted in the solid water phantom of 110 (length) × 15 (width) × 8 (depth) cm^3^ in which dummy bone marrow structure (PTV) and organs at risks such as lung, kidney, and body subtracting from target were contoured (Figure 1).

The prescription of 18 Gy/3fx was used for planning simulation to cover 80% of PTV. We used the jaw size, modulation factor and pitch of 5.0 cm, 3.5, and 0.20, respectively, so that the treatment time was over 1800 seconds.

For dose verification, two ion chambers at 2 cm depth were put at 27.5 cm and at 72.5 cm from tip of the phantom at SAD = 85 cm (Figure 1). Two EDR-2 films (Kodak, Rochester, NY, USA) were also placed in a line in the phantom from 25 cm to 85 cm from the tip. Film analysis was performed in delivery quality assurance workstation and the comparison of longitudinal profile and gamma analysis with the criteria of 3 mm/3% between planned and measured dose were performed.

## Results and discussion

Although the new DCS has been offered to the current tomotherapy machine since 2011, there are still a number of tomotherapy machines without DCS in clinics all over the world. In the present study, we investigated the very long time output variation in this type of tomotherapy machine which is known to have output variation, typically about ± 2%, due to the absence of a dose servo system [12,13]. Since the treatment plans are based on a constant output, a variation between planned and delivery could result. For example, Francois et al. reported that the output in rotation during 800 seconds looked very unstable just before target change [9]. The output variation is therefore concerned especially in the treatment of TBI or TMI which requires about 30 minutes of beam-on time. To our knowledge, this is the first report that investigated the very long time variation assuming TMI delivery.

**Figure 2:**
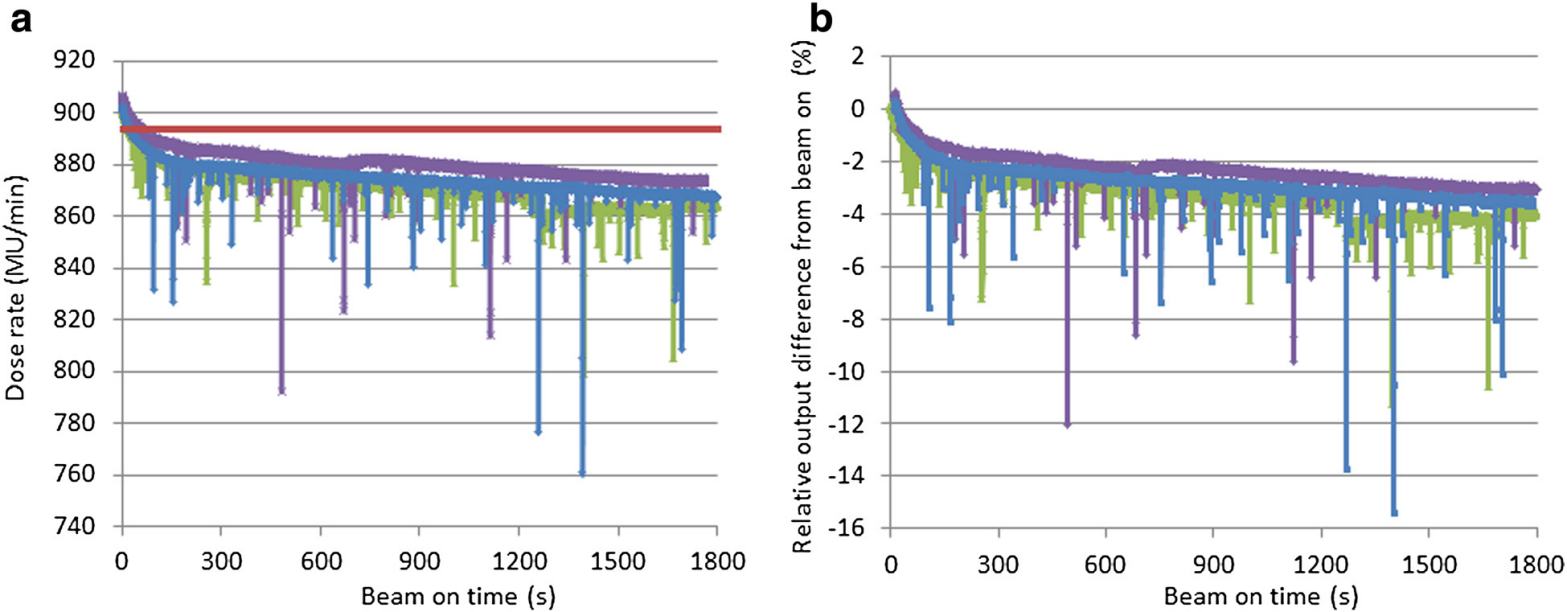
**Static output variation during 30 minutes irradiation. (a)** Dose rate by time, **(b)** Relative output difference from start. Red line indicates the reference output which is input in treatment planning system. Blue, green, purple lines show the 1^st^, 2^nd^, 3^rd^ measurements, respectively.

Figure 2 (a) shows the long time output variation measurements of the static beam mode. These were repeated 3 times. The red line indicates the reference dose rate of 894 MU/min that was given in the tomotherapy treatment planning system in our institute. The output gradually decreased during 30 min with an average (±SD) of −2.9% ± 0.2%, -3.4% ± 0.3%, and −3.4% ± 0.3% at 10 min, 20 min, and 30 min, respectively (Figure 2 (b)). The output variation was up to 5%. On the other hand, static output variations from reference dose rate during 30 min averaged −2.0% ± 0.4%, -2.4% ± 0.5%, and −2.8% ± 0.5% at 10 min, 20 min, and 30 min, respectively. The steep output drop-off was observed within 3 minutes from beam-on.

**Figure 3:**
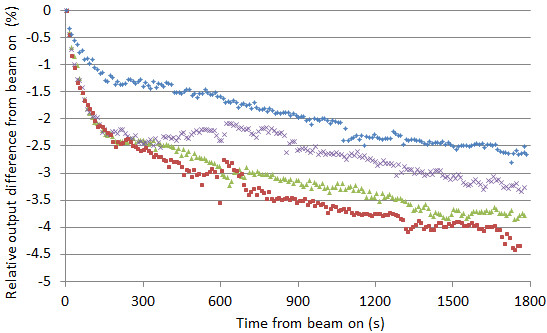
**Rotational output variation during 30 minutes’ with the gantry rotation speed of 10 sec/rotation using the cylindrical phantom.** The ion chamber reading was collected every 0.1 sec. Four lines with different colors show repeated measurements. Because the rotational output oscillates, the average values of each cycle were calculated and plotted. The dose rate at time 0 was 903 MU/min.

The same tendency was observed in rotational beam delivery. Figure 3 shows the long time output variation measurements for a rotational radiation delivery of 10 seconds/rotation gantry period. Measurements were repeated 4 times. The output gradually decreased with an average (±SD) of −2.5% ± 0.7%, -3.1% ± 0.7%, and, -3.5% ± 0.8% at 10 min, 20 min, and 30 min, respectively. The output decrease from beam-on was up to 4.5%. Again, the steep output decrease occurred within the first 3 minutes from beam-on.

**Figure 4:**
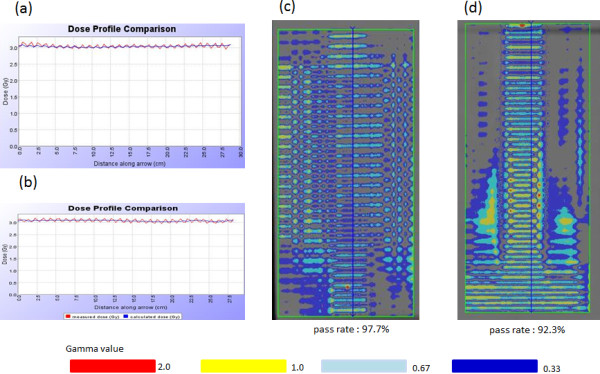
**Verification of TMI planning with EDR-2 films. (a)** Longitudinal profile in cranial and **(b)** caudal sides, **(c)** Gamma distribution of cranial and **(d)** caudal sides.

Although up to 5% of long time output variations were observed, the TMI planning dose agreed well with the film and the ion chamber measurements. Figures 4 (a) and (b) show the longitudinal profiles of head and foot regions in the model TMI planning, respectively. No remarkable dose differences were observed in both head and foot regions. Figure 4 (c) and (d) showed the gamma distribution with the criteria of 3 mm/3% in head and foot region, respectively. The pass rates were 97.7% and 92.2%, respectively. The ion chamber measurements in both head and foot regions agreed within 3% with the calculated dose.

In spite of continuous decrease in output during 30 minutes of irradiation, the impact of long TMI treatment time on delivered dose was small. One possible reason is that output decreased rapidly the first 3 minutes and then slowly in both static and rotational modes. In our institute, we calibrate the machine output at a dose rate of about 903 MU/min with the static beam at the depth of 1.5 cm in the field size of 40 cm (width) x 5 cm (length) at 85 cm SSD. Considering that the overall output dose fluctuation was 3% on average, then the delivered average dose rate was only 2% lower compared to the reference dose rate (894MU/min) in the treatment planning. Assuming it is applicable for rotational output variation, the magnitude of output variation from reference dose rate would be smaller in a 30 minutes’ TMI treatment. If larger output fluctuation is observed, then the use of a higher initial dose rate would reduce the impact on real TMI or TBI treatment delivery. However, we do not find any safety issue with the tomotherapy machine even without DCS.

In the tomotherapy machine, the treatment will be terminated if (i) the monitor chamber readings differ by more than 50% from their nominal rate for more than 3 seconds or (ii) the monitor chamber readings differ by more than 5% from their nominal rate for more than three consecutive rolling 10 seconds windows [12,13]. In a long treatment time therapy such as TBI and TMI, these kinds of interruptions should be avoided because it increases treatment time.

Duchateau et al. reported that output fluctuations in the imaging beam mode with an average imaging time of 173 seconds (range; 111–281 seconds) were between 5% and 9% during the acquisitions [8]. Although the treatment beam and imaging beam originate from the same source, the rotational variation in treatment beam mode is typically on the order of ± 2% [12,13]. Furthermore, it has been shown through simulated data that sinusoidal rotational variations on the order of ± 5% results in systematic dose uncertainty within ± 2% [10]. More recently, Staton et al. reported that simulation of 2% or 7% rotational output change results in less than 1%, or 2% for DVH values, respectively [11]. Our treatment plan verification results were therefore reasonable.

In conclusion, TMI delivery with helical tomotherapy without DCS does not pose a risk for significant deviations from the original treatment plan regardless of the output fluctuation during treatment. The magnitude of long time output drop-off may vary depending of machine issues of which the magnetron condition is critical. For example, replacement of old magnetron with new one may improve the long time output variation. In the TBI or TMI treatment with helical tomotherapy that does not have DCS, however, quality assurance of very long time output variation should be performed before the first treatment.

## Competing interest

The authors declare that they have no conflict of interest.

## Authors’ contributions

YT carried out all the experiments and wrote the article. SH conceived of this study, and participated in its design and coordination and helped to draft the manuscript. Both authors read and approved the final manuscript.
